# Two Cases of Early Viability, and an Interesting Case of Premature Birth

**Published:** 1853-09

**Authors:** 

**Affiliations:** Lecturer on Midwifery, McGill College, Physician to the University Lying-in-Hospital, &c.


### OBSTETRICS.

*Two cases of early viability, and an interesting case of premature birth* By M. McCulloch, M. D., M. R. C. S. L., Lecturer on Midwifery, McGill College, Physician to the University Lying-in-Hospital, &c.—Mrs. Henderson, aged 28, the wife of a grocer in this city, had been married about a year, and was attended by me in her first labor on the 13th February, 1849. She was delivered of a son whose weight was ascertained by his father and myself not to exceed thirty-six ounces, and his length, likewise accurately taken, was fourteen inches. He had some hair on his head, and cried as soon as he was born. He was wrapped in cotton wool, and within twenty-four hours sucked his mother's breasts without difficulty. Her nipples were well formed and she had already a sufficient supply of milk. On the 24th he weighed forty-seven ounces, and on the 13th March, when a month old, his weight was 3lbs. 11 ounces, and length 16 inches. On 13th April, I put him again into his father's scales, and found his weight to be 5lbs. 2 ounces, and length 17½ inches. Up to this date his health had been so uniformly good that he never needed any kind of medicine, and he was, in every respect, except size, a fine thriving child. Soon after the commencement of the third month he was unfortunately exposed to cold, and died of inflammation of the lungs, after being ill about a week.

The mother of this child could not remember the date of the last appearance of the catamenia nor when she quickened, but thought she became pregnant about the first of July, and if there was no error regarding this date, her son had been in utero seven months and thirteen days, and there must have been some check to his uterine progress, but if we take nto consideration the favorable state of his health and daily increase in bulk after birth, and his immature weight when two months old, I think his mother may have been mistaken regarding the first of July, and that it is more probable the duration of his previous existence did not exceed the end of the sixth month.

Mrs. Galbraith miscarried on the 28th of January. She became again pregnant before the catamenia returned, and was delivered by me on the 23d of July—175 days, or twenty-five weeks after the miscarriage. The mother refused to allow the child to be weighed, but I ascertained its length to be thirteen inches. It is probable that its weight was less than two pounds, and that the duration of pregnancy did not exceed twenty-two weeks. Notwithstanding, it survived three hours, and its cries were so loud and frequent during the first forty minutes as to astonish every one present.

Mrs. ——, the wife of a former steward of the Montreal General Hospital, the mother of several children, had a child born within 6 months and 14 days of the possible commencement of pregnancy. Previous to this period, she had resided some months out of town and menstruated regularly. She was the patient of a medical friend, and I had an opportunity of seeing the child about six weeks after its birth, when it was still very small, and I had no doubt of its being a satisfactory case of early viability. It survived, and is now upwards of 10 years of age: It took the breast well at birth. The testes descended, two months after birth, accompanied by a loop of intestine, producing scrotal hernia, which was radically cured by the use of a truss at the age of 18 months.—*Montreal Monthly Journal of Medicine & Surgery.*

